# Routes to improving the reliability of low level DNA analysis using real-time PCR

**DOI:** 10.1186/1472-6750-6-33

**Published:** 2006-07-06

**Authors:** Stephen LR Ellison, Claire A English, Malcolm J Burns, Jacquie T Keer

**Affiliations:** 1 Analytical Technology, LGC Limited, Teddington, TW11 0LY, UK

## Abstract

**Background:**

Accurate quantification of DNA using quantitative real-time PCR at low levels is increasingly important for clinical, environmental and forensic applications. At low concentration levels (here referring to under 100 target copies) DNA quantification is sensitive to losses during preparation, and suffers from appreciable valid non-detection rates for sampling reasons. This paper reports studies on a real-time quantitative PCR assay targeting a region of the human SRY gene over a concentration range of 0.5 to 1000 target copies. The effects of different sample preparation and calibration methods on quantitative accuracy were investigated.

**Results:**

At very low target concentrations of 0.5–10 genome equivalents (g.e.) eliminating any replicates within each DNA standard concentration with no measurable signal (non-detects) compromised calibration. Improved calibration could be achieved by eliminating all calibration replicates for any calibration standard concentration with non-detects ('elimination by sample'). Test samples also showed positive bias if non-detects were removed prior to averaging; less biased results were obtained by converting to concentration, including non-detects as zero concentration, and averaging all values.

Tube plastic proved to have a strongly significant effect on DNA quantitation at low levels (*p *= 1.8 × 10^-4^). At low concentrations (under 10 g.e.), results for assays prepared in standard plastic were reduced by about 50% compared to the low-retention plastic. Preparation solution (carrier DNA or stabiliser) was not found to have a significant effect in this study.

Detection probabilities were calculated using logistic regression. Logistic regression over large concentration ranges proved sensitive to non-detected replicate reactions due to amplification failure at high concentrations; the effect could be reduced by regression against log (concentration) or, better, by eliminating invalid responses.

**Conclusion:**

Use of low-retention plastic tubes is advised for quantification of DNA solutions at levels below 100 g.e. For low-level calibration using linear least squares, it is better to eliminate the entire replicate group for any standard that shows non-detects reasonably attributable to sampling effects than to either eliminate non-detects or to assign arbitrary high Ct values. In calculating concentrations for low-level test samples with non-detects, concentrations should be calculated for each replicate, zero concentration assigned to non-detects, and all resulting concentration values averaged. Logistic regression is a useful method of estimating detection probability at low DNA concentrations.

## Background

Accurate measurement of DNA using quantitative real-time PCR (Q-PCR) techniques is fundamental to many molecular tests with clinical [1], environmental [2], and forensic applications [3]. Quantitative DNA analysis at high target concentration is reproducible [4], but demand for increasing sensitivity requires low levels of DNA to be measured with equal reliability. This presents a challenge to analysts as there are numerous problems associated with trace detection and quantification, yet a range of sectors are increasingly reliant on low level analyses. Clinically there is growing interest in quantifying levels of circulating nucleic acids for a range of applications including diagnosis and monitoring of cancer patients [5], prognosis for victims of trauma [6] and non-invasive prenatal diagnosis [7]. Similarly, forensic laboratories are often presented with extremely small amounts of material crucial to a legal case; accurate analysis must then be carried out at the first attempt as often there is insufficient sample for repeated investigations [8]. Additionally at an international level, legislation governing the limits for genetically modified organisms (GMOs) in foods necessitates the use of sensitive and accurate methods to detect and quantify trace levels of GMO ingredients [9].

Quantification is initially reliant upon having suitable standards in the appropriate concentration range and of the same quality as the samples being analysed. The chosen DNA standard must itself be quantified either by spectrophotometer, which has limitations [10] or with an intercalating dye such as PicoGreen^® ^[11,12] before being used to prepare standard curves. There are currently no certified reference materials available for DNA quantification, so accuracy depends on the way in which individual laboratories measure and prepare the DNA standard. Dilution protocols involved in constructing standard curves are recognised as contributing to reduced reliability at low template concentrations [13,14] and there are several causes for this. Firstly the stochastic distribution of molecules means that at very low copy number a sampling error can be introduced when pipetting aliquots of DNA. Measurement variability at low DNA concentration has been demonstrated by the observation that the confidence intervals (representing the measurement uncertainty) associated with amplification from small initial copy numbers of template are much greater than those with large initial copy numbers [15]. Increasing the number of replicate analyses performed on low concentration standards and samples can mitigate inaccuracy caused by this inherent variability, and improve analytical sensitivity [16]. A second issue is the apparent lability of DNA when stored in solution at low concentrations, which can significantly affect the accuracy of quantification if dilution series are stored for prolonged periods prior to use. A report by Teo *et al*. [4] highlighted the short time scale over which fluctuations in the concentration of standard solutions can occur, due to DNA binding to untreated microcentrifuge tube walls. This is of particular significance at low concentrations when there are only a small number of molecules present and has ramifications for the quantification of unknown samples. The result of all these factors is that large measurement uncertainty is associated with trace-level DNA quantification [10] and the performance characteristics of PCR-based assays at low target concentrations are ill defined.

The measured instrument response for Q- PCR is the Ct value, which is the amplification cycle at which the fluorescent signal from each reaction increases above a nominal threshold value. An additional problem when working with Q-PCR at trace level is the occurrence of valid negative reactions (here referred to as non-detects), which do not have a quantitatively meaningful Ct value, as the fluorescent signal does not reach the threshold level during the course of the reaction. These are valid negative reactions, reflecting true absence of analyte because of sampling variability at low target concentration. Such negative results are either arbitrarily awarded a value equal to the number of amplification cycles run (here a Ct of 55), or are not allocated a measurable value. Assigning a value equal to the total number of cycles run causes difficulties in subsequent data analysis as the value will vary dependent on cycling parameters, and is often identified as an outlier value by normal statistical analysis. The non-normal distribution of results around zero generated by real-time data has also been noted by other researchers [17]. A common practice is to exclude all such negative reactions entirely from the analysis, whether obtained from unknown samples or standards. However at trace levels the non-amplification provides accurate information about the low concentration analytes, reflecting both the distribution of molecules and sampling effects. Selective omission of negative observations in these circumstances can generally be expected to lead to biased quantification.

The purpose of this investigation was to identify measures to increase the reliability of low level DNA quantification and detection by Q-PCR. Firstly, we performed a series of experiments to assess practical interventions for DNA standard preparation, which could potentially reduce the variability of low concentration DNA solutions and make construction of calibration curves more accurate. This involved preparing DNA dilutions in different types of plastic ware and with a range of diluents. Using the results from these experiments, we explored data handling approaches for standard curve construction and sample quantification, with particular attention to the treatment of valid negative observations. Finally, routes to assessing detection probability for characterising analytical sensitivity and performance in qualitative applications were examined. Logistic regression was applied to determine the detection probability of analyte at a particular concentration, and calculations based on binomial statistics developed to aid prediction of the degree of replication required to achieve a particular level of sensitivity.

## Results and discussion

### Effect of standard DNA preparation on quantification

The effect of different standard preparation conditions was assessed by analysis of *z*-scores for estimated concentration, calculated as described below (see Methods, where the rationale for *z*-scoring is also presented). The estimated concentrations were calculated using calibration curves calculated using the exclusion-by-sample method described below (see Construction of calibration curves, below). Note that *z*-scores are expected to have mean 0 and variance 1. In the scheme used here, negative *z*-scores correspond to lower than average concentration compared to the average for the group. When normally distributed, 95% of *z*-scores are expected to be within approximately ± 2.

One-way ANOVA of the *z*-scores for between-plate differences showed a strongly significant effect (*p*-value = 6.1 × 10^-7^) (confirmed by a Kruskal-Wallis test (K-W) *p*-value of 3.4 × 10^-6^). This presented a choice; re-calculate concentrations by fitting plate by plate, or, equivalently with no Condition/Plate interaction term, take advantage of the balanced design and treat Plate as a blocking factor. Blocking by plate is the preferred option, as it naturally adjusts for the Plate effect on the residual degrees of freedom, which would otherwise be slightly overestimated. Graphical inspection indicated that the Plate effect was not large enough to raise concerns about level-dependent differences in variance, and two-way ANOVA confirmed that there was no significant interaction (at the 95% level of confidence) between Plate and Condition, so the analysis reported here used blocking by plate. The relatively small Plate effect also permitted continued application of the K-W test as a follow-up test, as the Plate effect has the practical effect of increasing within-group variance and consequently will tend to produce more conservative *p*-values for the K-W test. To check the effect of separate construction of calibration curves for each plate, the analysis was repeated after separate calibration by plate, with no effect on the conclusions and only marginal effects on *p*-values. We also examined the effect of simply scaling by robust standard deviation at each concentration without *z*-scoring (while treating Concentration as an error term); again, this had no effect on the conclusions.

"Condition" (Table 1) was a combination of tube plastic (low-retention and standard) and solution (tissue culture water, stabilising solution, and carrier DNA). Initial analysis sought a general Condition effect. ANOVA for Condition effects, blocked by Plate, gave a significant between-condition effect (*p *= 0.007), supported by the K-W test *p*-value of 0.03 (similar, in fact, to the *p*-value of 0.01 for one-way ANOVA without blocking). Homogeneity of *z*-score variance across Conditions was poor, but not grossly so; Levene's test gave a *p*-value of 0.035, warranting caution, but unlikely to prejudice the ANOVA results greatly.

**Figure 1:**
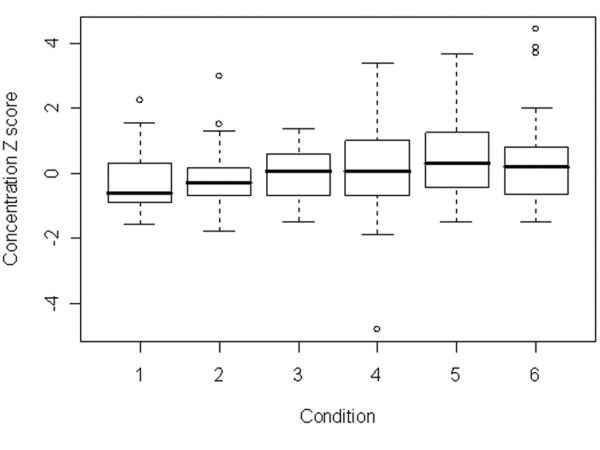
**Boxplot of concentration z score by Condition**. Base: All observations for concentrations 0.5–1000. See Table 1 for preparative condition details. Briefly: Conditions 1–3 are standard plastic tubes and conditions 4–6 low-retention plastic. Pairs (1,4), (2,5) and 3,6) correspond to water, stabilising solution and carrier DNA solutions respectively.

Inspection of the *z*-scores by Condition (Figure 1) showed that conditions 1–3 appeared low compared to Conditions 4–6. This was potentially important since Conditions 1–3 used standard plastic tubes, and 4–6 used low-retention tubes. Otherwise, no specific group stands out. However, given a significant Condition effect, Plastic and Solution effects were investigated further.

**Figure 2:**
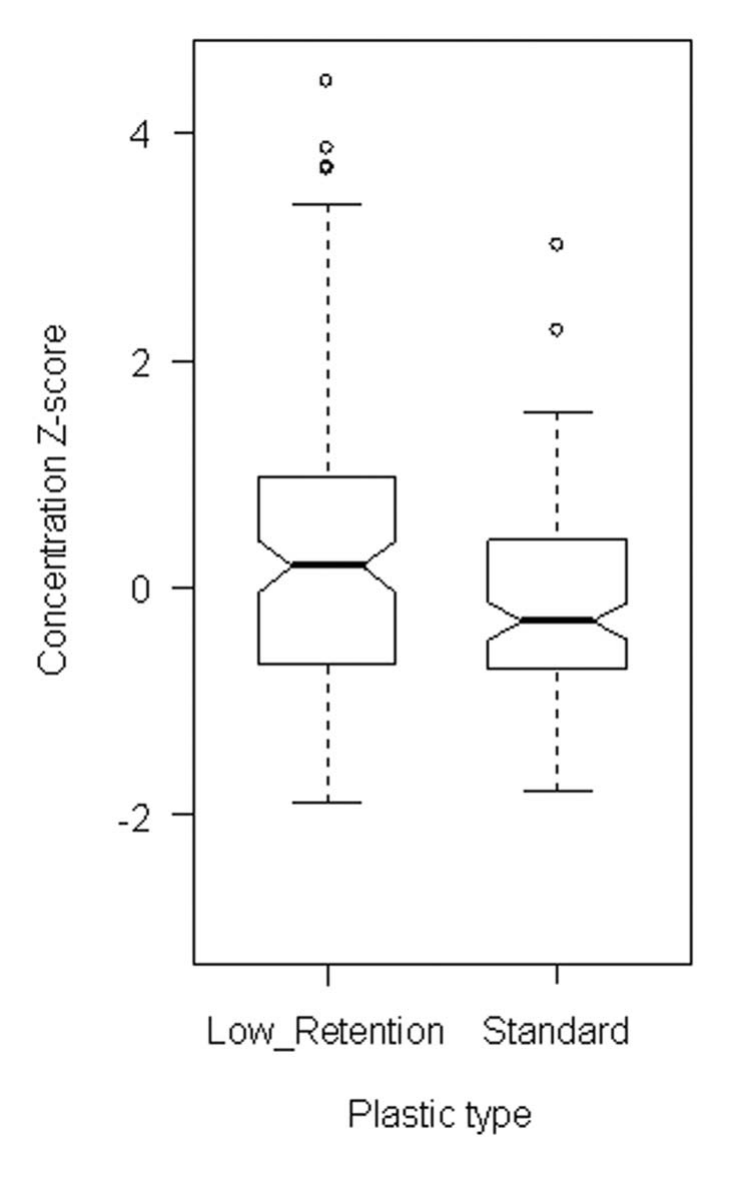
**Effect of tube plastic on DNA quantitation**. Notched box plot of concentration z-core against plastic type. Notches show approximate 95% confidence intervals for the median; non-overlapping notches (as here) indicate significant difference. Base: All observations for concentrations 0.5–1000.

**Figure 3:**
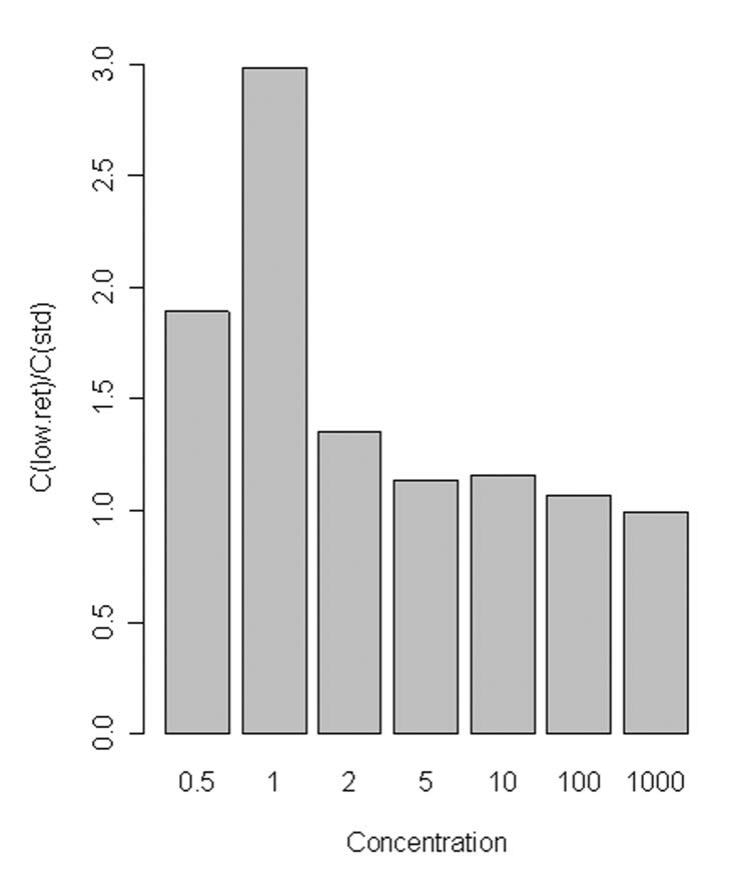
**Effect of low retention plastic by concentration**. The figure shows the ratio of mean predicted concentrations (C) for low-retention and standard plastic tubes (the ratio is mean(low ret.)/mean(standard)), grouped by nominal concentration (copy number). Note that low-concentration groups include non-amplification events; the ratios partly reflect the reduced incidence of non-amplifications in low-retention tubes. Means were calculated from all observations for concentrations 0.5–1000.

Tube plastic proved to be strongly significant (*p *= 1.8 × 10^-4^) (K-W *p*-value = 0.002). Inspection showed, as might be expected, that low-retention plastic ware gave higher observed concentrations than standard plastic (Figure 2). The effect was particularly evident at low concentrations, and essentially negligible over 100 g.e., as shown in Figure 3. Despite some possible differences on visual inspection (Figure 1), (for example, conditions 1 and 4 are both low and are for a particular solution), the Solution effect proved insignificant at the 95% level of confidence (*p *= 0.51 for one-way ANOVA supported by a K-W test *p*-value of 0.49). These observations were essentially unchanged on performing 2-way ANOVA for Plastic and Solution effects, and there was no significant interaction between the two factors.

These results have practical relevance for performance of trace DNA measurements. The significant plate effect was not unexpected, as inter-plate differences are common in Q-PCR work. In general, calibration plate by plate is to be recommended; this is, however, normal practice and no additional recommendation is necessary. The strong effect of low-retention plastic is, however, likely to be important for low-level quantification. Based on Figure 3, standard plastic tubes may give a two-fold reduction in apparent concentration at low levels, compared to low-retention plastic. This result is in agreement with a study by Teo *et al *[4], which concluded that siliconised tubes reduced inconsistencies caused by abstraction of DNA from solution to the walls of untreated microcentrifuge tubes. Teo *et al *also found no significant effect on using carrier DNA for standard solutions using the Lightcycler. Given the likelihood that the significant effect of low-retention plastic is related to loss of DNA in standard tubes, and the improved accuracy at low concentrations, it seems safe to recommend the use of low-retention plastic when working with solutions at low DNA concentrations, here below 100 g.e.

### Construction of calibration curves at low DNA concentration

At higher DNA concentrations (for this assay, at and over 100 target copies per reaction), negative results almost invariably indicate amplification failure and can justifiably be removed as invalid observations. At low DNA concentrations, however, particularly below about 10 target copies per reaction, sampling issues mean that a significant proportion of the replicate reactions genuinely do not contain any target. The question then arises of how best to calibrate, when data contain a significant fraction of valid negative responses.

**Figure 4:**
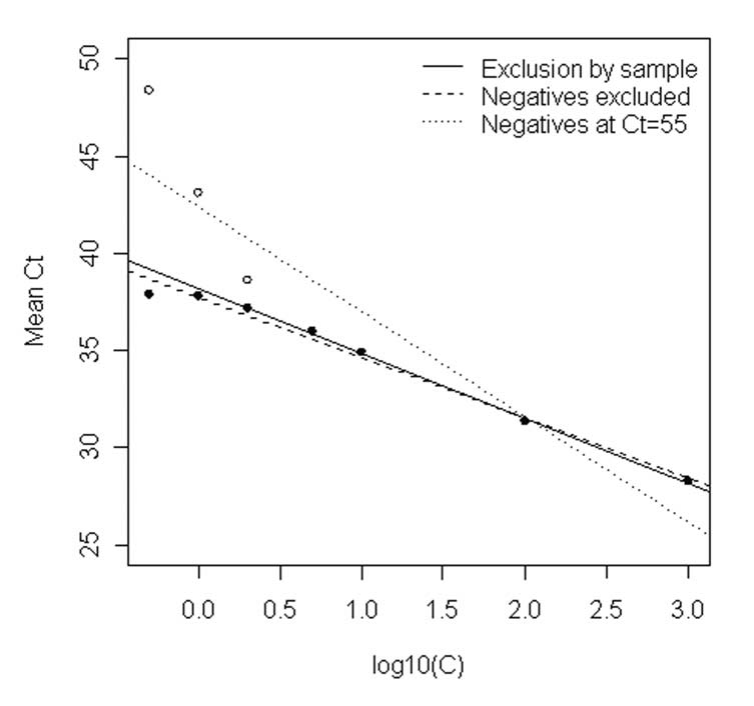
**Calibration for low level DNA quantification**. Base: All valid observations. The figure shows mean values at each concentration with a) (filled circles ●) negative observations excluded; b) (open circles o) all values including negative observations taken as Ct = 55 (the means coincide at log_10_(C)>0.5). Regression lines are the exclusion-by-sample regression (solid line), the line through means with negative observations excluded (dashed line) and the line through means with negative observations set to Ct = 55 (dotted line).

Exclusion of the negative observations alone, as at high concentrations, introduces a marked downward bias in the average Ct value derived for low-concentration standards. This is most clearly seen in the appearance of an upper limit or 'plateau' for mean Ct values at low concentrations (Figure 4, filled circles). This plateau effectively represents Ct value for amplification of a single target molecule. This plateau precludes discrimination between samples of different low concentrations. Further, the negative bias has adverse effects on the complete calibration curve, the more so as the biased points are at one extreme of the line and consequently introduce a rotational bias; compare the solid and dashed lines in Figure 4. Thus at very low concentrations, omission of individual reactions with no measurable signal prevents discrimination at low levels and causes inaccuracy at higher concentrations in the same assay. Bias introduced by poor reproducibility of low level calibrators has also recently been reported by Lai *et al *[18].

One alternative to omitting negative observations is to include the non-amplification events occurring in low level standards in the calculation of the mean Ct values used for constructing the standard curve, using the (arbitrary) limiting value (open circles in Figure 4). The change in probability of negative responses then leads to an expectation of non-linear, but monotonic, increase in mean Ct with reducing concentration, in principle permitting discrimination between very low level samples. Linear regression is clearly inappropriate (Figure 4, dotted line), but interpolation using an empirical curve fit to such data has been previously reported as successful [19]. However, this approach has several weaknesses. It is very sensitive to the proportion of negative responses, so depends on high levels of replication to guarantee near-monotonic change in observed mean value. The interpolation is arbitrary and cannot readily be generalised to wide concentration ranges. The coefficients change with arbitrary changes in the limiting value used. Finally, the mean Ct value arises from a mixture of quantitative data and arbitrarily coded qualitative responses, making the error distribution complex, seriously compromising the validity of a least-squares approach to interpolation, and making prediction uncertainties very hard to obtain. These shortcomings make the approach unsuitable for routine use.

The approach taken in the present study has therefore been to remove *all *data for any standard compromised by valid negative responses (in this paper this will be referred to as "exclusion-by-sample"). The rationale is simply that this eliminates selection bias in the calibration. It is also very simple to implement, so can be used by a routine laboratory with existing software. The results are shown in Figure 4, which shows the exclusion-by-sample line for all data (*y *= 37.7 - 3.09*x*) as the solid line.

The approach has the obvious disadvantages of extrapolation to obtain low-concentration values and some loss of precision. However, extrapolation is valid as long as the model is sound. The log-linear model is well supported by the underlying physical process, and effective method validation should in any case confirm linearity over the range of interest before undertaking analysis. The loss of precision is not very marked; recall that the lower concentration samples typically show high variance and should in any case be down-weighted. Prediction precision can also be improved by extending the calibration data to *higher *concentrations as long as continued linearity is demonstrated, and also, in principle, by increasing replication towards the extremes of the calibration range (with caution at the lower concentrations, as amplification failure becomes more likely with increased replication). The most serious practical disadvantage is therefore that it may not be clear in advance which samples will be excluded, resulting in wasted effort and resources and, sometimes, precluding reliable calibration entirely if insufficient high-concentration samples are included. There is also some risk of excluding samples that show negative responses for other reasons.

These latter disadvantages can be ameliorated relatively easily. Experience or validation studies will show where the risk of exclusion is sufficiently low to warrant acquisition of data; this is discussed further below in connection with detection probabilities. Extending the concentration range upward to provide more precise extrapolation is straightforward, and involves no additional effort; the lowest concentration standards that are at high risk of exclusion can be replaced by higher-concentration standards. It is, however, important to identify and remove aberrant observations at higher concentrations (such as the single failure at 100 g.e. in the present study). Objective criteria are important when assessing suspect observations, and there is little or no standard practice corresponding to outlier detection in Q-PCR work. It is accordingly premature to make firm recommendations here. It may, however, be helpful to note that where a 'true' negative has probability of occurrence of 1% or less, removal of negative observations prior to analysis would have little adverse impact on calibration.

Exclusion by sample is nonetheless quite drastic and risks eliminating a proportion of valid calibrant data, as well as incurring uncertainty associated with extrapolation. In the longer term, therefore, there is a need to investigate approaches that use all the valid data and accommodate the negative observations without introducing bias. For example, maximum likelihood fitting is in principle capable of handling arbitrary error distributions and can accommodate censored data. Robust regression [20] or median-based methods could be employed to reduce the adverse impact of arbitrary assignment of high values, yet still allow inclusion of all the observations in construction of calibration curves. The ideal regression method would also take into account the fact that part of the error arises from variability in the actual copy number, that is, that there is significant uncertainty in the independent as well as the dependent variable. In the mean time, in the absence of such methods we recommend inclusion of only those standards in which all replicates yield measurable values to ensure that bias is avoided in construction of calibration curves. If higher concentration standards have negative reactions that are identified as outliers, the outliers can still justifiably be excluded.

### Quantification of unknown samples

In contrast to the situation in constructing calibration curves, exclusion of Ct values by sample is not a viable option when an unknown sample is analysed. Fortunately, calculations are in any case best carried out in the concentration domain, where one has the option of treating negative observations as zero concentration. Two methods for handling negative observations (that is, reactions failing to yield a measurable Ct value) in the calculation of unknown sample concentration were accordingly compared to determine the most accurate approach. The comparison used the replicate data from the lower concentration standards prepared in low-retention plastic only, with a calibration model based only on standards from low-retention plastic. In one approach, negative reactions were excluded from the calculation entirely, the remaining Ct values converted to concentration and the average concentration taken. In the other, negative amplifications were included in the quantification by assigning a concentration value of zero for the observation, and again the resulting individual concentration estimates were averaged. The first of these approaches – excluding negative reactions – is expected to introduce positive bias due to selection for positive reactions only; increasingly so as the incidence of true negatives increases. By contrast, although there may be other sources of bias, assigning a concentration value of zero to non-detects is expected to introduce negligible additional bias as long as the distribution of Ct for true positive runs has negligible density above the Ct threshold.

The results are shown in Table 2. There is a slight positive bias throughout, perhaps because of slight departures from linearity, but the results obtained on excluding reactions with Ct = 55 clearly gave consistently higher mean sample concentrations than assigning zero concentration to all such values. At 5 target copies and above both methods gave the same result as all reactions yielded measurable values. The results obtained on setting negative responses to zero concentration were all closer to the nominal concentration for the standards. This showed that, as expected, excluding negative results leads to undesirable positive bias; more importantly, the bias so induced can be considerable for high proportions of failure.

The practical implication is that in quantification of unknown samples at low copy number, valid negative observations (i.e. those valid observations that have a Ct equal to the number of cycles run) should be treated as samples with zero observed concentration. Thus, to calculate the concentration of a particular sample, the mean of the concentration estimates obtained from Ct values via the calibration curve should be calculated, including the negative observations as zero concentration. This allows all available information on the sample to be included in the final result, as also detailed recently by Zimmermann *et al *[21]. As Table 2 demonstrates, this provides better estimates of the unknown sample concentration. The caveat is that negative observations arising through amplification failure rather than genuine absence of target should be excluded or otherwise accommodated. As in the case of calibration curve construction, appropriate criteria should be applied; again, we note that exclusion of observations on the basis of low probability of occurrence (e.g. 1% or below) is unlikely to lead to appreciable bias but will avoid the adverse effects of amplification failure on higher-concentration samples.

### Detection probability and detection limits

Estimates of detection probability at low levels are important for several reasons. An understanding of detection probability is useful in determining the expected frequency of detection for a particular concentration analyte, when performing a defined number of replicate analyses. It can assist in deciding whether or not to reject amplification failures at low levels, and aid realistic interpretation of assay results. Performance characteristics such as limit of detection (sensitivity) for qualitative PCR-based methods also rely on probability of detection. Finally, understanding the detection probability for a single amplification can assist in deciding optimal replicate numbers and in choosing minimum concentrations for reasonable confidence of detection and/or avoidance of detection failures.

To estimate the number of replicate reactions required to achieve a particular number of successful amplifications (at a given probability), the binomial distribution is appropriate. The binomial distribution describes the probability of *n*_*s *_successes in *n*_*t *_trials (i.e. replicates) with a probability of success (per trial) of *p*_*s*_. Fortunately, recourse to the distribution is unnecessary for the special cases of *n*_*s *_= *n*_*t *_and *n*_*s *_= 0, which are the only cases required for our purposes. The probability of *n*_*t *_successes in *n*_*t *_trials (no failures) is psnt
 MathType@MTEF@5@5@+=feaafiart1ev1aaatCvAUfKttLearuWrP9MDH5MBPbIqV92AaeXatLxBI9gBaebbnrfifHhDYfgasaacH8akY=wiFfYdH8Gipec8Eeeu0xXdbba9frFj0=OqFfea0dXdd9vqai=hGuQ8kuc9pgc9s8qqaq=dirpe0xb9q8qiLsFr0=vr0=vr0dc8meaabaqaciaacaGaaeqabaqabeGadaaakeaacqWGWbaCdaqhaaWcbaGaee4CamhabaGaemOBa42aaSbaaWqaaiabbsha0bqabaaaaaaa@32B0@; that of no successes is (1−ps)nt
 MathType@MTEF@5@5@+=feaafiart1ev1aaatCvAUfKttLearuWrP9MDH5MBPbIqV92AaeXatLxBI9gBaebbnrfifHhDYfgasaacH8akY=wiFfYdH8Gipec8Eeeu0xXdbba9frFj0=OqFfea0dXdd9vqai=hGuQ8kuc9pgc9s8qqaq=dirpe0xb9q8qiLsFr0=vr0=vr0dc8meaabaqaciaacaGaaeqabaqabeGadaaakeaadaqadiqaaiabigdaXiabgkHiTiabdchaWnaaBaaaleaacqqGZbWCaeqaaaGccaGLOaGaayzkaaWaaWbaaSqabeaacqWGUbGBdaWgaaadbaGaeeiDaqhabeaaaaaaaa@364E@. The probability of one or more successes (positives) is then 1-(1−ps)nt
 MathType@MTEF@5@5@+=feaafiart1ev1aaatCvAUfKttLearuWrP9MDH5MBPbIqV92AaeXatLxBI9gBaebbnrfifHhDYfgasaacH8akY=wiFfYdH8Gipec8Eeeu0xXdbba9frFj0=OqFfea0dXdd9vqai=hGuQ8kuc9pgc9s8qqaq=dirpe0xb9q8qiLsFr0=vr0=vr0dc8meaabaqaciaacaGaaeqabaqabeGadaaakeaadaqadiqaaiabigdaXiabgkHiTiabdchaWnaaBaaaleaacqqGZbWCaeqaaaGccaGLOaGaayzkaaWaaWbaaSqabeaacqWGUbGBdaWgaaadbaGaeeiDaqhabeaaaaaaaa@364E@. The number *n*_min _of replicates required to ensure a minimum probability *p*_min _of at least one positive is then given by

*n*_min _= *ceiling*(log(1-*p*_min_)/log(1-*p*_s_))     (1)

where *ceiling*(*x*) is the nearest integer greater than or equal to *x*.

The calculation requires an estimate of the probability of detection at the concentration(s) of interest, usually derived from validation or calibration standard data. Given sufficient replication, the observed fraction of positive results at each concentration provides a direct estimate of probability for the particular concentration (a positive result is considered as the detection of target in a sample, thus any reaction with a Ct value lower than 55 in this assay). Use of experimental counts is straightforward. It is, however, somewhat sensitive to random failures, leaves little possibility of interpolation to find probabilities at intermediate concentrations, and typically requires a relatively high number of replicates at each concentration to provide reliable probability estimates.

Modelling the probability offers a useful alternative. The detection data is binomial (Positive or Negative), and logistic regression a widely accepted method of modelling binomial responses. We therefore applied logistic regression to the data set above to determine the probability of target detection across the concentration range.

**Figure 5:**
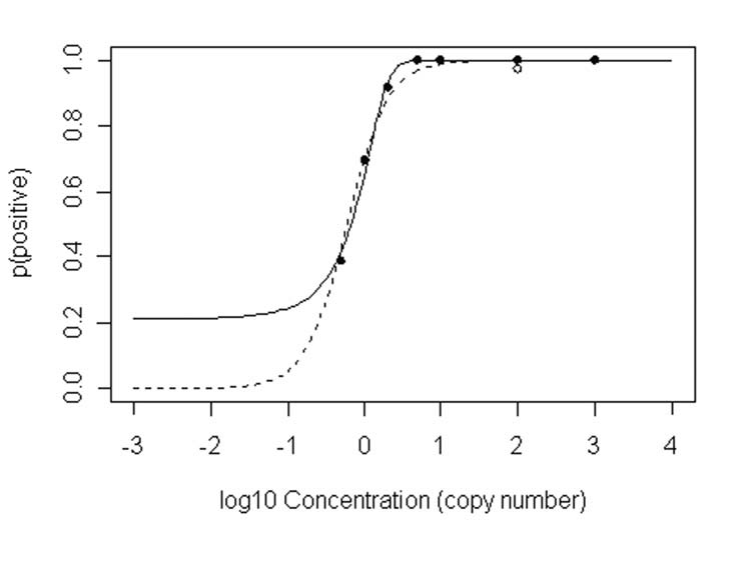
**Logistic regression of detection probability**. Logistic regression of a) (solid line) p(positive) vs. concentration C with false negative at log_10_(C) omitted; b) (dashed line) p(positive) vs. log_10_(C) with false negative included. Solid points show fraction of positives at each concentration with the false negative omitted; the open circle shows the calculated fraction at log_10_(C) with the false negative included. The apparent lower limit to the solid curve is an artefact of plotting on the log_10 _axis.

Logistic regression can be biased by errors at extreme concentrations, and PCR data often shows concentrations spanning several orders of magnitude. Here, the initial fit against concentration C (omitting C = 0) was relatively poor due to the amplification failure at 100 g.e. (residual deviance 183.02 on 250 degrees of freedom, with AIC* of 187.02). With the amplification failure at 100 g.e. omitted, the fit in the concentration domain improved dramatically (residual deviance: 113.57 on 249 degrees of freedom, with AIC improved to 117.57); this curve was p(positive) = 1/(1+exp(-(-1.322+1.960C))). The fitted curve is shown in Figure 5 (solid line). Using the fitted probabilities in the binomial calculation, replicate numbers at the observed concentrations are 6, 3, and 2 for 0.5, 1 and 2 copy numbers, and 1 thereafter, for 95% probability of at least one positive. This compares well with calculations based on observed fractions.

Ideally, aberrant values should be excluded prior to applying logistic regression, as here. If that cannot be done with confidence (for example, for lack of sufficient replication at any one concentration) we suggest fitting in the log domain for PCR data to reduce the weight associated with failures at high concentration. Fitting against log_10_(C) provided a good fit to the low-concentration data even when the amplification failure at 100 g.e. was included (residual deviance 133.53 on 250 degrees of freedom, with AIC of 137.53; this should be compared with the substantially poorer AIC of 187.02 for the initial fit to concentration C, above). This line was p(positive) = 1/(1+exp(-(0.854+3.7506*log_10_(C)))); the curve is shown as a dashed line in Figure 5. If anything, this curve appears more realistic on visual inspection, in that it correctly predicts very low probability at very low concentrations, where the concentration domain model apparently predict significant probability of positives. Predicted replication numbers using this curve were, however, identical to those previously found.

Some caution is necessary in modelling probability. Using a log scale for the independent variable, as here, is essentially an arbitrary choice; although Ct is expected to be linear in log_10_(C), there is no *ab initio *reason to expect p(positive) to be determined by log_10_(C) rather than C, and the logarithmic spacing of concentrations is driven by practical choice, not necessity. Further, logistic regression itself, though a widely accepted and successful methodology for binary response modelling, the logistic model is only one possible model for binary responses [22]. Wherever it is used, the fitted model needs to be checked for quality of fit before being applied to interpolation, and the most appropriate variant selected. Even then, extrapolation to extreme probabilities is particularly dangerous unless there is very strong prior evidence that the model applies.

Overall, both observed counts and logistic regression provided broadly similar conclusions in this case. Both were somewhat sensitive to aberrant values; logistic regression excessively so in the concentration domain. In the log domain, logistic regression was less affected. The advantages of the logistic regression approach include the general smoothing effect of model fitting; the use of the entire data set in fitting the model, in principle decreasing the number of replicate measurements required for reliable prediction at any one concentration; the possibility of interpolation; and the possibility of estimating uncertainties in the probability estimates from the uncertainties in fitted coefficients.

Logistic regression can also be applied to the estimation of analytical sensitivity. In the present study, for example, it was found that the concentration providing 50% probability of detection for a single observation (sometimes taken as an estimate of detection limit, or sensitivity, for biological assays) corresponded, using the log_10_(C) curve, to a value of log10(C) = -0.2275 and an actual concentration of 0.592 copies, indicating an impressive likelihood of detectable amplification of a single copy. A similar application, using probit regression, was recently reported by Zimmermann *et al *[21]; in our study, we examined probit regression in the log_10_(C) domain for comparison, and found that it gave a closely similar result to the logistic regression, as expected. Further, logistic regression can assist in estimating the lowest concentration at which amplification failure rate remains acceptable, avoiding wasted resources when employing the recommended 'exclusion by sample' method in calibration. For example, 95% probability of six positives in six replicates requires an individual run success probability of 0.991 (using the binomial calculation above, we see that 0.991^6 ^= 0.95); based on the log-domain logistic regression for our data, the individual success probability of 0.991 would be expected at a concentration of about 11 g.e. Note, however, that lower concentrations also have quite high success probabilities (e.g. 0.83 for 5 g.e.); it is therefore not particularly surprising that we obtained 100% successful amplifications down to 5 g.e.

## Conclusion

Tube plastic had a strong effect on measured DNA concentration at concentrations below 100 g.e., and it is recommended that low-retention plastics be used for DNA quantification below this level. Neither stabilising solution nor carrier DNA had a significant effect under the conditions of this study.

For low-level calibration, it is better to eliminate all Ct values in a replicate group for any standard concentration that shows non-detects reasonably attributable (over 1% probability based on binomial statistics) to sampling effects, than to eliminate only the non-detects. There is, however, a need for further work to establish improved fitting methods in the low-concentration region

In calculating concentrations for low-level test samples that show appreciable non-detects, concentrations should be calculated for each replicate, zero concentration assigned to non-detects, and all resulting concentration values averaged. Average Ct values should not be used to estimate concentrations.

Logistic regression was found to be a useful method of estimating detection probability at low DNA concentrations, provided that measures are taken to remove, or reduce the effect of, invalid non-amplifications at high concentrations.

## Methods

### Calibration standard preparation

A set of experiments was designed to test the influence of different calibration standard preparation variables on the sensitivity of low level DNA detection using Q-PCR. Standards were prepared by serial dilution of human male genomic DNA to investigate the effects of using low retention plastics, stabilising solution and carrier DNA. Six different conditions were employed in preparing the standards as shown in Table 1. Regular or low retention plastic microtubes were used with water, a commercially produced stabilising solution (Cambio, UK) or carrier DNA solution (herring sperm DNA, Sigma, UK, at 10 μg/ml) as diluent for the calibration standards.

Standards at eight concentrations were prepared from a DNA stock solution (Promega, UK) at a concentration of 5000 genome equivalents (g.e.) per μl. The stock was quantified with PicoGreen^® ^intercalating dye (Molecular Probes, Netherlands) on a fluorescence plate reader, using a commercially purified DNA Standard Solution (Cambio, UK) prepared gravimetrically. Results were expressed as genome equivalents based on the calculated concentration, using the conversion factor of 6.6 pg of DNA per diploid genome. The stock DNA was also measured on an ND-1000 spectrophotometer (NanoDrop Technologies) to verify the PicoGreen^® ^value. Standard curves in the range 1000 to 0.5 g.e./μl, and negative controls (containing only the appropriate diluent) were prepared in volumes of between 200–1000 μl. Previous work demonstrated that amplification sensitivity and accuracy improved for low concentration standards prepared in relatively large volumes (200–1000 μl), compared to smaller ones (<50 μl, data not shown). Each point of the standard curve was assayed in duplicate to enable the 6 different standard preparation conditions to be compared directly on one reaction plate. The whole assay was repeated 3 times to produce replicate sets of data for comparison, and Ct value was the measurand used to assess all standard DNA preparation conditions. In order to complete analysis of 3 plates in one day using the same analyst, the dilutions were prepared late the day before and stored at +4°C overnight.

### Q- PCR assay

The Q- PCR was performed on the PRISM^® ^7700 Sequence Detector System (Applied Biosystems, UK) using the 5'-nuclease assay with a dual labelled fluorogenic "TaqMan" probe. All oligonucleotides were designed according to the guidelines recommended by Applied Biosystems. A coding region of the male specific SRY gene was chosen as the amplification target and the primer and probe sequences were as follows:

Forward primer: 5'CGATCAGAGGCGCAAGATG3'.

Reverse primer: 5'TGGTATCCCAGCTGCTTGCT3'.

Probe: 5'-VIC-TCTAGAGAATCCCAGAATGCGAAACTCAGAGA-TAMRA-3'

The PCR for each 25 μl reaction was composed of: Forward primer 450 nM (Sigma, UK), reverse primer 450 nM (Sigma, UK), TaqMan probe 225 nM (Applied Biosystems, UK), 1 × Excite PCR mix (Biogene, UK) and 1 μl of DNA in a total volume of 25 μl. Each PCR was run for 55 cycles (50°C, 2 min, UNG activation; 95°C, 10 min, denaturation, followed by 55 cycles of 95°C, 5 sec and 60°C, 1 min, amplification). Initial treatment of the raw data was carried out using the Applied Biosystems SDS software.

Contamination was minimised by preparing reaction mixtures in a dedicated clean room with reagents aliquoted into single use volumes. The 1 × reaction mix contained Uracil-N-Glycosylase (UNG) to prevent carry-over contamination between PCR reactions and the DNA was added in a separate template addition area. The quality of each standard curve was assessed by checking the negative controls for amplification signals. If any false positive signals were detected, data from the affected curve was discarded and the run repeated (which was necessary for one 8-point standard curve in this set of experiments).

### Statistical methods

The raw Ct values [see Additional file 1] were not very amenable to analysis for preparation effects in production of the DNA standards. The variances differed substantially between concentrations (Levene's test gave a *p*-value of 5.9 × 10^-14^), and the values assigned to non-amplifications (Ct = 55) were essentially arbitrary, generating arbitrary non-normality and effectively placing the data on an ordinal rather than interval scale. These difficulties were addressed by, first, working in the concentration domain and then *z*-scoring by concentration. The procedure was as follows: All valid log(concentration)/Ct values were fitted using the exclusion-by-sample method. Sample concentrations were calculated from the fit coefficients. Samples with Ct = 55 were set to zero concentration. The resulting concentration estimates were *z*-scored by group, using robust estimates of location and scale (Huber proposal 2, following ([23]). Specifically, the concentrations were converted to *z*-scores using *z*_*ij *_= (*x*_*ij *_- x^
 MathType@MTEF@5@5@+=feaafiart1ev1aaatCvAUfKttLearuWrP9MDH5MBPbIqV92AaeXatLxBI9gBaebbnrfifHhDYfgasaacH8akY=wiFfYdH8Gipec8Eeeu0xXdbba9frFj0=OqFfea0dXdd9vqai=hGuQ8kuc9pgc9s8qqaq=dirpe0xb9q8qiLsFr0=vr0=vr0dc8meaabaqaciaacaGaaeqabaqabeGadaaakeaacuWG4baEgaqcaaaa@2E35@_*j*_)/s^
 MathType@MTEF@5@5@+=feaafiart1ev1aaatCvAUfKttLearuWrP9MDH5MBPbIqV92AaeXatLxBI9gBaebbnrfifHhDYfgasaacH8akY=wiFfYdH8Gipec8Eeeu0xXdbba9frFj0=OqFfea0dXdd9vqai=hGuQ8kuc9pgc9s8qqaq=dirpe0xb9q8qiLsFr0=vr0=vr0dc8meaabaqaciaacaGaaeqabaqabeGadaaakeaacuWGZbWCgaqcaaaa@2E2B@_*j *_where x^
 MathType@MTEF@5@5@+=feaafiart1ev1aaatCvAUfKttLearuWrP9MDH5MBPbIqV92AaeXatLxBI9gBaebbnrfifHhDYfgasaacH8akY=wiFfYdH8Gipec8Eeeu0xXdbba9frFj0=OqFfea0dXdd9vqai=hGuQ8kuc9pgc9s8qqaq=dirpe0xb9q8qiLsFr0=vr0=vr0dc8meaabaqaciaacaGaaeqabaqabeGadaaakeaacuWG4baEgaqcaaaa@2E35@_*j *_is the Huber estimate of location for concentration *j*, and s^
 MathType@MTEF@5@5@+=feaafiart1ev1aaatCvAUfKttLearuWrP9MDH5MBPbIqV92AaeXatLxBI9gBaebbnrfifHhDYfgasaacH8akY=wiFfYdH8Gipec8Eeeu0xXdbba9frFj0=OqFfea0dXdd9vqai=hGuQ8kuc9pgc9s8qqaq=dirpe0xb9q8qiLsFr0=vr0=vr0dc8meaabaqaciaacaGaaeqabaqabeGadaaakeaacuWGZbWCgaqcaaaa@2E2B@_*j *_the corresponding robust standard deviation. *z*-Scoring by concentration was chosen instead of more general transformation or scaling because a) the dependence of variance on concentration was not straightforward and b) as well as standardising the variance, *z*-scoring removed the primary effect of concentration which would otherwise have to be controlled for in testing the effects of interest. The reason for using robust scaling parameters in z-scoring was to reduce any adverse effects of extreme. Levene's test showed, as expected, that the *z*-score variances were essentially identical across concentrations.

The scores remained non-normally distributed for some low concentrations, but the overall distribution was not grossly non-normal. To confirm that the findings were not critically dependent on normality assumptions, any significant one-way ANOVA was followed up by a Kruskal-Wallis (K-W) test. The Kruskal-Wallis tests confirmed the ANOVA result in every case.

The aberrant negative observation at 100 g.e. was excluded from the analysis.

In addition, logistic regression was used to predict the probability of detection of a positive signal at all target concentrations. Detection positives were judged as reactions with Ct<50.

### Software

Statistical analysis was carried out in R version 2.1.0 [24]

## Authors' contributions

CAE carried out the experimental work and contributed to the manuscript. SLRE drafted the final manuscript, advised on regression methods and carried out the statistical analysis for condition effects and logistic regression. MJB assisted in study design and carried out preliminary statistical analysis. JTK managed the project and had input at all stages of the study. All authors read and approved the final manuscript.

## Note

* AIC = Akaike information criterion

## Supplementary Material

Additional File 1 The data shows the different Q-PCR reactions conditions set up, the concentration of DNA in each reaction and the resulting Ct value achieved from the corresponding Q-PCR reaction. Click here for file

## Figures and Tables

**Table 1:** Preparation of DNA standards. Details of the conditions used in preparation of the serial dilution DNA solutions for standard curve construction.

**Method**	**DNA Standard preparation condition**
1	Standard plastics (1.5 ml microtubes, Alpha). Water as diluent (Tissue culture water, Sigma, UK).
2	Standard plastics. Stabilising solution as diluent (Stabilising component for DNA standards, Cambio, UK).
3	Standard plastics. Carrier DNA as diluent (Herring Sperm DNA, Sigma, UK @ 10 μg/ml).
4	Low retention plastics (Non-stick hydrophobic microtubes 1.5 ml, Alpha). Water as diluent.
5	Low retention plastics. Stabilising solution as diluent.
6	Low retention plastics. Carrier DNA as diluent.

**Table 2:** Comparison of sample quantification methods.

**Concentration/target copies μl**^**-1**^	**Number of negative reactions**^**Note 1**^	**Measured concentration**	
		
		**Ct of 55 included (as zero concentration)/target copies μl**^**-1**^	**Ct of 55 excluded/target copies μl**^**-1**^
0.5	10	0.66	1.48
1.0	2	1.68	1.89
2.0	3	2.51	3.02
5.0	0	5.28	5.28
10.0	0	10.90	10.90

Note 1: Number of reactions (of 18 replicates) failing to reach fluorescence threshold, and with Ct set to 55. Results showing the difference in sample concentration determined by either including or excluding amplification reactions with no measurable Ct value. The calibration curve was calculated from the low-retention data only, using the exclusion-by-sample method to remove samples with missing values, and the interpolated concentrations calculated using the low-retention plastic values only.
